# Supplementary material for: FLNC Expression Level Influences the Activity of TEAD-YAP/TAZ Signaling
Source: Genes (Basel). 2020 Nov 13;11(11):1343. doi: 10.3390/genes11111343 (PMC7696573; doi:10.3390/genes11111343)
Supplement: Supplementary file 1 [file genes-11-01343-s001.pdf]

Supplementary table S1. Oligonucleotide sequences used for CRISPR/Cas9 plasmid cloning, qPCR and sequencing.

Gene	Oligonucleotide sequence (5'-3')	Application
Myh1 (Forw)	GCTGAGAGAAGCTACCACATT	qPCR
Myh1 (Rev)	ACAAAGGCGTAGTCGTATGG	qPCR
Myh4 (Forw)	CAACAGTGCAGAGCAGGGAAG	qPCR
Myh4 (Rev)	GGCCATGTCCTCAATCTTGTCGTAC	qPCR
*Mymk (*Forw).	CCTGTGATGGGCCTGGTTTGTC	qPCR
*Mymk (*Rev)	GGTTCATCAAAGTCGGCCAGTGC	qPCR
*Myog (*Forw)	GAGACATCCCCCTATTTCTACCA	qPCR
*Myog (*Rev)	GCTCAGTCCGCTCATAGCC	qPCR
*Ctgf (*Forw)	CCCTAGCTGCCTACCGACT	qPCR
*Ctgf (*Rev)	GTAACTCGGGTGGAGATGCC	qPCR
*Cyr61 (*Forw)	CTGAAGAGGCTTCCTGTCTTT	qPCR
*Cyr61 (*Rev)	GTGGTCTGAACGATGCATTTC	qPCR
Flna (Forw)	GCTAAAGGTCACTGTAAAGGGT	qPCR
Flna (Rev)	TTCACCTCAAACGGACTTCGAC	qPCR
Flnb (Forw)	GGGGAAAGTAACCTGCGTGA	qPCR
Flnb (Rev)	CATGGCTGTGACTTCCCCAT	qPCR
*Gapdh (*Forw)	GGATCTGACGTGCCGCCTG	qPCR
*Gapdh (*Rev)	GAAGGTGGAAGAGTGGGAGTTGC	qPCR
Flnc_guide sense	CACCGTATTCGGACGCCTCCGGCCT	CRISPR plasmid cloning
Flnc_guide antisense	AAACAGGCCGGAGGCGTCCGAATAC	CRISPR plasmid cloning
Scramble_guide sense	CACCGGTGCGAATACGCCACGCGAT	CRISPR plasmid cloning
Scramble_guide antisense	AAACATCGCGTGGCGTATTCGCACC	CRISPR plasmid cloning
U6	GAGGGCCTATTTCCCATGATTC	Sequencing
*Flnc_exon1 (*Forw)	GCGCTAACGAAGTCTCCGA	Sequencing, allele cloning
*Flnc_exon1 (*Rev)	TTGAGATGCTCGTTGCACCA	Sequencing, allele cloning

Supplementary Figure S1

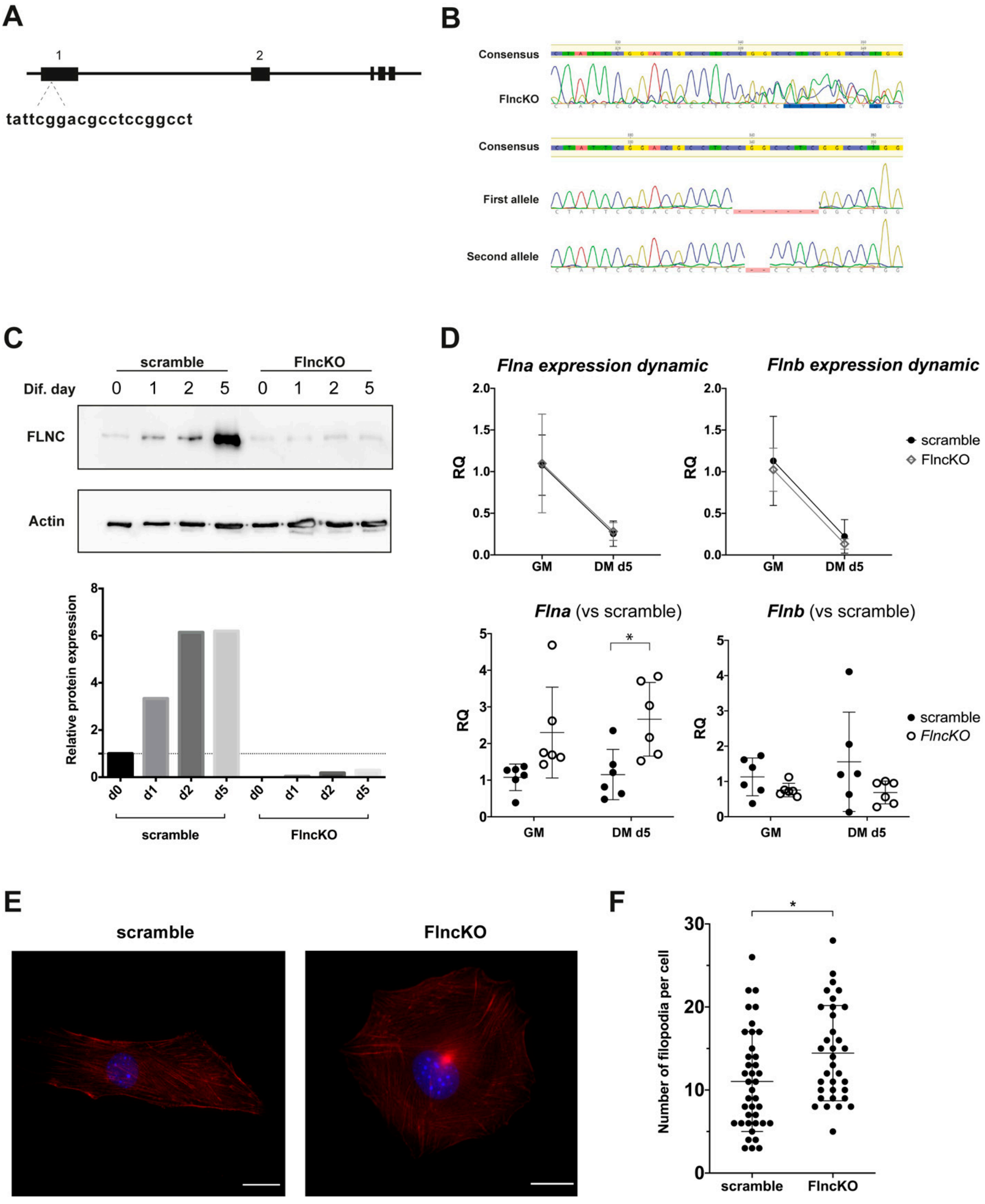

Figure S1. Generation of FLNC-deficient C2C12 cell line using CRISPR/Cas9 technique.

(A) GuideRNA-driven CRISPR/Cas9 genome editing performed within the first exon of mouse *Flnc*. (B) Sanger sequencing of total cell DNA represents introduction of deletions 3 nucleotides upstream to PAM-site. TA-allele cloning revealed the presence of two different deletions in each allele (2 nt and 7 nt), no WT alleles was observed after screening of at least 20 TA-clones. (C) Western Blotting of *FlncKO* cells showed loss of FLNC in undifferentiated myoblasts with slight increase of FLNC during myogenic differentiation compared to a scramble cell line (control). (D) Expression dynamics of *Flna* and *Flnb* during myogenic differentiation in scramble control and *FlncKO* cells(upper panel), and changes of Flna and Flnb expression in *FlncKO* cells comparing to scramble control (lower panel). (E) Representative images of scramble control and FlncKO undifferentiated myoblasts stained with Phalloidin Rhodamine. (D) Quantification of filopodia sites per cell in undifferentiated FlncKO and scramble control myoblasts. GM – growth media, DM d5 – 5 days in differentiation media.*$p < 0.05$. Scale bar 20 μm.
